# Random Mutational Analysis Targeting Residue K^155^ within the Transmembrane β-Hairpin of the Mosquitocidal Mpp46Ab Toxin

**DOI:** 10.3390/biology12121481

**Published:** 2023-12-01

**Authors:** Midoka Miyazaki, Mami Asakura, Toru Ide, Tohru Hayakawa

**Affiliations:** Graduate School of Interdisciplinary Science and Engineering in Health Systems, Okayama University, 3-1-1 Tsushima-naka, Kita-ku, Okayama 700-8530, Japan; pyi831r1@s.okayama-u.ac.jp (M.M.); asakura@cc.okayama-u.ac.jp (M.A.); ide@okayama-u.ac.jp (T.I.)

**Keywords:** mosquito-larvicidal toxin, *Bacillus thuringiensis* TK-E6, *Culex pipiens* mosquito larvae, site-directed mutagenesis, electrophysiologic analysis

## Abstract

**Simple Summary:**

Mpp46Ab is a mosquito-larvicidal pore-forming toxin derived from *Bacillus thuringiensis* TK-E6. We previously constructed Mpp46Ab mutants targeting charged amino acid residues within the transmembrane β-hairpin and revealed an apparent correlation between the channel-pore cation selectivity and mosquito-larvicidal activity of Mpp46Ab. In particular, residue K^155^ was thought to be a good target to improve Mpp46Ab, as replacement of K^155^ with glutamic acid (K155E) resulted in the increased cation selectivity of the channel pores and increased mosquito-larvicidal activity. In the present study, we assessed the importance of the amino acid at residue 155 by constructing an Mpp46Ab mutant library in which residue K^155^ was randomly replaced with other amino acids. Upon mutagenesis and following primary screening using *Culex pipiens* mosquito larvae, we obtained 15 mutants in addition to the wild type. Bioassays using purified proteins revealed that the toxicity of the K155E and K155I mutants was significantly higher than that of the wild-type toxin. Interestingly, the cation selectivity of K155I channel pores was significantly increased, as previously observed for K155E channel pores, suggesting that the two mutations induce a similar conformational change in the region involving the ion selectivity of the Mpp46Ab channel pores.

**Abstract:**

Mpp46Ab is a mosquito-larvicidal pore-forming toxin derived from *Bacillus thuringiensis* TK-E6. Pore formation is believed to be a central mode of Mpp46Ab action, and the cation selectivity of the channel pores, in particular, is closely related to its mosquito-larvicidal activity. In the present study, we constructed a mutant library in which residue K^155^ within the transmembrane β-hairpin was randomly replaced with other amino acid residues. Upon mutagenesis and following primary screening using *Culex pipiens* mosquito larvae, we obtained 15 mutants in addition to the wild-type toxin. Bioassays using purified proteins revealed that two mutants, K155E and K155I, exhibited toxicity significantly higher than that of the wild-type toxin. Although increased cation selectivity was previously reported for K155E channel pores, we demonstrated in the present study that the cation selectivity of K155I channel pores was also significantly increased. Considering the characteristics of the amino acids, the charge of residue 155 may not directly affect the cation selectivity of Mpp46Ab channel pores. Replacement of K^155^ with glutamic acid or isoleucine may induce a similar conformational change in the region associated with the ion selectivity of the Mpp46Ab channel pores. Mutagenesis targeting the transmembrane β-hairpin may be an effective strategy for enhancing the ion permeability of the channel pores and the resulting mosquito-larvicidal activity of Mpp46Ab.

## 1. Introduction

Mpp46Ab is a toxin derived from *Bacillus thuringiensis* strain TK-E6 and formerly designated “Cry46Ab” in previous nomenclature [1]. Research has shown that Mpp46Ab is highly cytotoxic to human leukemic T cells (MOLT-4 and Jurkat) but has virtually no effect on human embryonic kidney cells (HEK293) [2]. Mpp46Ab was thus designated parasporin-2Ab as a member of the toxin family that exhibits preferential cytotoxicity against human cancer cells. In addition to the cytotoxicity, Mpp46Ab reportedly exhibits insecticidal activity against *Culex pipiens* mosquito larvae [3]. Mpp46Ab is thus a broad-spectrum toxin, exhibiting both selective cytotoxicity against human leukemic T cells and insecticidal activity against *C. pipiens* mosquito larvae.

Mpp46Ab exhibits significant homology (84% identity) to Mpp46Aa (previously known as Cry46Aa or parasporin-2Aa) from *B. thuringiensis* strain A1547 [2,4]. Mpp46Aa is a member of parasporin and is highly cytotoxic to hepatocellular carcinoma cells (HepG2), colon cancer cells (CACO-2), and leukemic T cells (MOLT-4, Jerkat and HL-60) [5]. A three-dimensional structural model of Mpp46Aa constructed based on X-ray crystallography analysis indicated significant structural similarity with aerolysin-type β-pore–forming toxins (β-PFTs) [6]. Considering this similarity, Mpp46Ab is also thought to function as a PFT, and indeed produces ion-permeable channel pores in artificial lipid bilayers [3]. The channel pores formed by Mpp46Ab are cation-selective (K^+^ > Cl^−^) and show some cation preference (K^+^ > Na^+^, K^+^ > Ca^2+^, and Ca^2+^ > Na^+^) [7]. 

**Figure 1:**
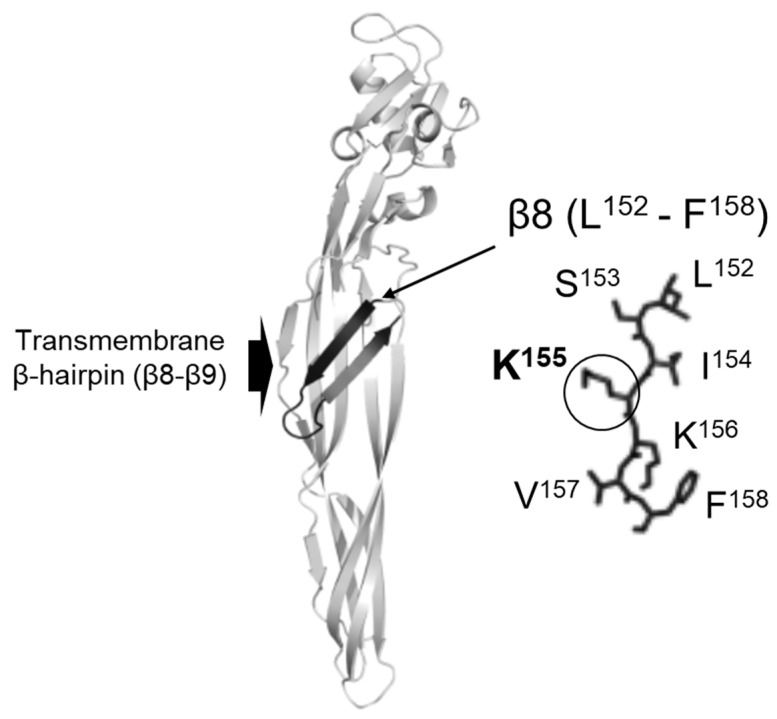
Structure of the Mpp46Ab molecule and transmembrane β-hairpin (β8–β9). The three-dimensional structure of Mpp46Ab was constructed using SWISS-MODEL [15,16] with the Mpp46Aa PDB code (2ztb). Positions and amino acid residues in the transmembrane β8 sheet are indicated.

The middle domain of Mpp46Ab has β-hairpin structure (β8-β9) that spans residues L^152^ to T^168^ (Figure 1). Mpp46Ab and Mpp46Aa share identical amino acid sequences within the β-hairpin region [2,4]. The β-hairpin structure is generally composed of an alternating pattern of polar and hydrophobic amino acid residues and have been identified in many aerolysin-type β-PFTs, such as aerolysin [8], staphylococcal α-toxin [9], ε-toxin from *Clostridium perfringens* [10], and mosquito-larvicidal Bin toxin from *Lysinibacillus sphaericus* [11]. According to the pore-formation model of aerolysin, toxin inserts the β-hairpin structure into the target cell membrane and rearranges into a transmembrane β-barrel [12,13,14]. 

Pore formation is believed to be a central mode of Mpp46Ab action, and the ion permeability of the channel pores should, in particular, be closely related to its mosquito-larvicidal activity. We previously speculated that the charged amino acid residues within the β-hairpin line the lumen of Mpp46Ab channel pores and that the electrical environment generated by the charged residues affects the ion permeability of the channel pores. We constructed a series of Mpp46Ab mutants in which one of the charged residues in the β-hairpin was replaced with an oppositely charged residue, and the effect of the replacement on both ion permeability of the channel pores and resulting mosquito-larvicidal activity was then investigated [17]. A significant increase in cation-selectivity was observed on the channel pores formed by mutant K155E, along with increased toxicity. In contrast, the selectivity of channel pores formed by the mutants E159K and E163K was reduced, and the mutants exhibited decreased toxicity [17]. This result suggested that channel pore cation selectivity is a major determinant of the mosquito-larvicidal activity of Mpp46Ab and that cation selectivity can be controlled via mutagenesis targeting the transmembrane β-hairpin region.

In general, application of insecticidal toxins is always accompanied by the risk of selecting insecticide resistance in larval mosquito populations. To overcome this obstacle, it is desired to develop an insecticidal toxin with higher activity. In the present study, we speculated that the residue K^155^ within the β-hairpin would, in particular, be a good target for regulating the ion permeability of the channel pores and resultant mosquito-larvicidal activity of Mpp46Ab. We constructed an Mpp46Ab mutant library in which residue K^155^ was randomly replaced with other residues. Mpp46Ab mutants exhibiting significant toxicity were screened using a simplified bioassay employing *Culex pipiens* mosquito larvae, and these mutants were then identified by DNA sequencing analysis. To clarify the relationship between channel pore cation selectivity and associated insecticidal activity, the selected Cry46Ab mutants were subjected to ion-selectivity measurements using planar lipid bilayers.

## 2. Materials and Methods

### 2.1. Construction of the Mpp46Ab Mutant Library

Residue K^155^ within the transmembrane β-hairpin of Mpp46Ab (Figure 1) was randomly replaced with other residues. The random mutations were introduced via site-directed mutagenesis as described previously [17]. PCR was performed using KOD One^®^ PCR Master Mix (Toyobo Co., LTD. Life science department, Osaka, Japan) with a specific primer set (46Ab-K155r, AATCGACAGTTTAGTGGTAATTTT; 46Ab-K155rm-f, NNNAAAGTCTTTGAAATTGGTGGC). In the 46Ab-K155rm-f primer, the codon (AAA) encoding K^155^ was substituted with random triplet codes (NNN, N=A/C/G/T). The expression vector pGST-Cry46Ab-S1 [3] was used as a template for mutagenesis so that the Mpp46Ab mutants constructed in the present study could be expressed as glutathione *S*-transferase (GST) fusions. Upon self-ligation of the PCR-amplified DNA fragments, the ligate was transformed into *Escherichia coli* BL21. 

### 2.2. Primary Screening of Mpp46Ab Mutants

Mpp46Ab mutants exhibiting significant toxicity were screened from the mutant library using a bioassay employing *Culex pipiens* mosquito larvae (third instar). Mosquito larvae were reared from eggs supplied by the Research and Development Laboratory, Dainihon Jochugiku Co., Ltd. (Osaka, Japan). Bioassays were carried out in a 96-well microtiter plate with one larva per well.

For primary screening, cultures of *E. coli* cells expressing the Mpp46Ab mutant were prepared on a small scale. Briefly, *E. coli* clones generated by mutagenesis were randomly selected and precultured at 37 °C overnight (O/N) in 0.5 mL of LB medium containing 100 μg/mL ampicillin. Next, 0.1 mL of the O/N culture was transferred to 0.5 mL of newly prepared LB medium (containing 100 μg/mL ampicillin and 0.2 mM isopropyl-β-D-thiogalactopyranoside [IPTG]) and cultured at 30 °C for another 4 h to induce expression of the Mpp46Ab mutants. A 10-µL sample of each resultant culture (0.6 mL) was then added to a well containing 1 larva in 190 μL of water, and the sample was monitored for mortality 48 h after initiation. In the primary screening, 8 larvae were used to evaluate the toxicity of one mutant clone, and toxicity resulting in >30% mortality (>3/8 larvae) was considered significant. Selected Mpp46Ab mutants exhibiting significant toxicity were subjected to DNA sequencing analysis to identify the mutation.

### 2.3. Preparation of Mpp46Ab Mutants

Mpp46Ab wild-type and mutants expressed as GST fusions were purified as described previously [3]. Briefly, *E. coli* cells harboring the corresponding mutant plasmids were cultured in TB medium containing 100 μg/mL ampicillin until the OD_600_ reached 0.7. The expression of GST-Mpp46Ab mutants was induced by adding IPTG at a concentration of 0.1 mM, followed by culturing at 30 °C for another 4 h. GST-Mpp46Ab mutants were purified using glutathione-Sepharose 4B (GE Healthcare Bio-Sciences AB, Uppsala, Sweden). For electrophysiologic analyses, selected GST-Mpp46Ab mutants were activated by passage through an immobilized-trypsin column prepared as described previously [3]. Protein concentration was determined using a protein assay dye reagent (Bio-Rad Laboratories, Inc., Hercules, CA, USA) with bovine serum albumin as the standard, and purified proteins were analyzed by sodium dodecyl sulfate–polyacrylamide gel electrophoresis (SDS-PAGE) followed by visualization using Coomassie brilliant blue reagent (CBB stain one, Nacalai Tesque, Inc., Kyoto, Japan).

### 2.4. Measurement of Mosquito-Larvicidal Activity

The mosquito-larvicidal activity of the GST-Mpp46Ab mutants was estimated as described previously [3]. Briefly, 20 μg of purified GST-Mpp46Ab was adsorbed onto 1 mg of latex beads (0.8 mm diameter, Sigma-Aldrich Corp., St. Louis, MO, USA) for 1 h at room temperature and then administered to *C. pipiens* mosquito larvae (third instar) as a diet. Bioassays were carried out in a 96-well microtiter plate with 1 larva per well and 24 larvae per each concentration in an assay. Mortality was recorded at 48 h after toxin administration, and the 50% lethal dose (LC_50_) with 95% confidence interval was determined using a PROBIT analysis [18]. In the bioassay, purified GST used as a negative control exhibited no toxicity up to at least 2 µg/mL (data not shown). The experiments were repeated more than 5 times using the purified protein samples that were prepared independently. 

### 2.5. Electrophysiologic Analyses

Electrophysiologic analyses of the channel pores formed by Mpp46Ab mutants were performed as described previously [17]. Briefly, the experimental apparatus consisted of two chambers (upper, *cis* chamber; lower, *trans* chamber), such that the voltage in the solution of the *cis* chamber was connected to a patch-clamp amplifier by an Ag/AgCl electrode-defined membrane potential. The bottom of the *cis* chamber was a thin sheet of polyvinyl chloride with a small circular hole (φ200 µm) and a lipid bilayer was constructed by painting asolectin (phospholipids from soybean, Sigma-Aldrich Corp.) solution (40 mg/mL in n-decane) across the small hole. To constitute the Mpp46Ab channel pores in the lipid bilayer, asolectin liposomes were separately prepared in solution containing 1 M sucrose as described previously [7]. Activated Mpp46Ab protein was mixed with the liposome solution at a concentration of 25 μg/mL and then added to the solution in the *cis* chamber to facilitate fusion between the liposomes and the planar lipid bilayer.

To analyze the anion–cation selectivity of the channel pores, the channel currents were recorded in the presence of a 4-fold gradient of KCl across the lipid bilayer (600 mM KCl and 10 mM Tris-HCl [pH 8.0] in the *cis* chamber, 150 mM KCl and 10 mM Tris-HCl [pH 8.0] in the *trans* chamber). Data were analyzed using pClamp software ver. 11.1 (Axon Instruments, Roster City, CA, USA). The channel currents were recorded and plotted versus the corresponding applied voltage to generate current-voltage relationship graphs. The zero-current reversal potential (*V*_R_) was obtained as the X-intercept of the current-voltage relationship line. It was assumed that the movement of charged ions across the lipid bilayer equilibrates at the *V*_R_. The *V*_R_ values were then corrected by the values of the junction potential. The junction potential is generally due to different mobilities of ions at interfaces between different solutions and has previously been determined to be −0.4 mV under the conditions used in this study [19]. The anion–cation permeability ratio (*P*_K_/*P*_Cl_) was calculated using the Goldman–Hodgkin–Katz equation. Statistical significance was evaluated using Student’s *t* test.

## 3. Results

### 3.1. Construction and Screening of Mpp46Ab Mutants

We constructed Mpp46Ab mutants in which residue K^155^ was randomly replaced with other amino acids, and the resultant clones were subjected to primary screening using *C. pipiens* mosquito larvae. Specifically, 161 clones in total were randomly selected from the *E. coli* colonies generated by site-directed mutagenesis and were divided into 4 groups. In the screening for each group, clones expressing wild-type and mutant GST-Mpp46Ab were cultured on a small scale using 1.5-mL microtubes. After induction of GST-Mpp46Ab expression, the *E. coli* cultures were directly administered to mosquito larvae as a diet, and mortality was monitored after 48 h. To increase the efficiency, the expression level of Mpp46Ab mutants in *E. coli* cells were not assessed in the primary screening. 

**Figure 2:**
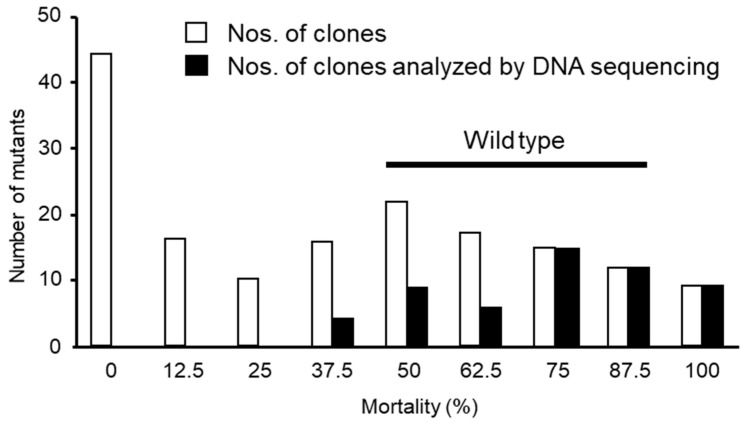
Summary of the primary screening assay using *C. pipiens* mosquito larvae. Open circles: mortality of mutant clones determined 48 h after administration. Filled circles: number of mutant clones selected for DNA sequencing analysis. Ninety-one of 161 randomly selected clones were positive (mortality >30%), and 55 clones were selected for DNA sequencing analysis from among clones exhibiting higher toxicity.

For *E. coli* cells expressing wild-type GST-Mpp46Ab, mortality rates ranged from 50 to 87.5% (Figure 2). We therefore tentatively considered clones producing mortality rates >30% as positive with significant toxicity. Among the 161 selected clones, 91 (57%) were positive, and the remaining 70 clones (43%) were negative (Figure 2). Fifty-five clones were further selected from the 91 positive clones, mainly those exhibiting higher toxicity, and then analyzed by DNA sequencing to determine the codon encoding amino acid residue 155 (Figure 2). 

Interestingly, the analyzed clones included a variety of mutants, and 16 of the 20 amino acids were represented at residue 155 of the mutants (Table 1). In brief, 8 clones were mutants in which a positively charged amino acid was at residue 155, as in wild-type Mpp46Ab. These included 3 K155R clones, 1 wild-type clone, and 4 K155H clones (Table 1). We also obtained 2 K155E clones in which positively charged K^155^ was replaced with negatively charged glutamic acid (Table 1). More interestingly, the most frequently isolated mutant was K155V (13 clones), in which positively charged K^155^ was replaced with highly hydrophobic valine (Table 1). Similar Mpp46Ab mutants such as K155F (6 clones), K155L (5 clones), and K155I (3 clones) were also identified with relatively high frequency. The nucleotide sequences of mutant *mpp46Ab* genes completely matched the sequence of wild-type Mpp46Ab except for the triplet code for residue 155. Neither stop codons nor unexpected mutations were found in the analyzed clones.

### 3.2. Mosquito-Larvicidal Activity of the Mpp46Ab Mutants

**Figure 3:**
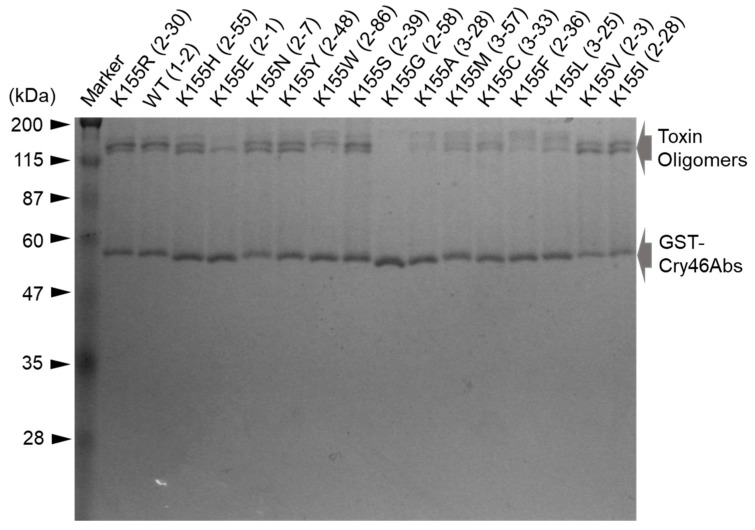
Electrophoretic analysis of wild-type and mutant Mpp46Abs. Wild-type and mutant GST-Mpp46Abs were purified using glutathione beads and analyzed by 10% SDS-PAGE. Five hundred nanograms of the purified protein was applied in each lane.

The mosquito-larvicidal activity of the Mpp46Ab mutants was investigated using GST-Mpp46Ab mutant toxin proteins prepared from the representative clones listed in Table 2. SDS-PAGE revealed that the molecular mass of the purified proteins was approximately 59 kDa similar to the expected mass of GST-Mpp46Ab (59.3 kDa) but varied slightly with each mutant (Figure 3). In addition, several protein bands with a higher molecular mass suggestive of oligomer formation were observed in most of the mutants (Figure 3). Although these protein bands were generally considered to be incomplete denaturation products, the protein bands did not disappear even under harsher condition (data not shown). There may be very rigid structure in the Mpp46Ab molecule and the replacement of K^155^ may affect the rigid structure. The purified proteins were then subjected to *C. pipiens* mosquito larvae bioassay, and the LC_50_ values 48 h after administration were calculated.

Overall, all GST-Mpp46Ab mutant toxin proteins showed significant toxicity against *C. pipiens* mosquito larvae. This suggested that the primary screening conducted in the present study was suitable for selecting mutants exhibiting significant toxicity. In brief, wild-type Mpp46Ab exhibited an LC_50_ value (95% confidence interval) of 0.51 (0.48–0.53) μg/mL (Table 2). The toxicity of the wild-type toxin observed in the present study was slightly higher than that (LC_50_ = 0.98 μg/mL) reported previously [17]. Among the Mpp46Ab mutants, the highest toxicity was observed for mutant K155E, followed by mutant K155I. The LC_50_ values (95% confidence intervals) of these mutants were 0.04 (0.03–0.05) and 0.09 (0.08–0.10) μg/mL, respectively (Table 2). The toxicity values of the remaining mutants were similar to or slightly lower than that of the wild-type toxin, with LC_50_ values ranging from 0.43 to 0.96 μg/mL (Table 2).

### 3.3. Anion–Cation Selectivity of the Channel Pores Formed by Selected Mpp46Ab Mutants

Three Mpp46Ab mutants (K155I, K155S, and K155R) were selected for electrophysiologic analysis using a planar lipid bilayer. Mutant K155I exhibited replacement of the positively charged K^155^ with highly hydrophobic isoleucine and showed toxicity significantly higher than that of the wild-type toxin (Table 2). In contrast, in the K155S and K155R mutants, positively charged K^155^ was replaced with uncharged polar serine and positively charged arginine, respectively. The toxicity of both mutants was lower than that of the wild-type toxin (Table 2). In the present study, a solution containing 29 kDa of the trypsin-activated Mpp46Ab mutants were desalted using an ultrafiltration device and then subjected to measurement. Measurements were repeated more than 6 times using activated Mpp46Ab mutants that were prepared independently. 

**Figure 4:**
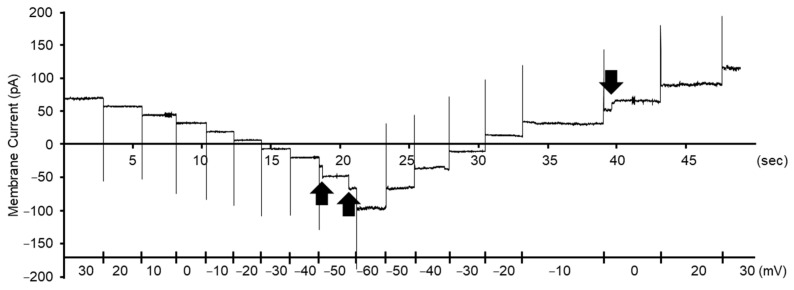
Representative segments of typical current traces for Mpp46ab mutant K155I recorded with a 4-fold gradient of KCl across the lipid bilayer. Current spikes suggesting fusion between a liposome with Mpp46Ab channel pores and a planer lipid bilayer constructed by the painting method are indicated by arrows. Applied voltage is shown on the lower axis.

After measurement initiation, we usually observed a current spike within several minutes. Similar current spikes were also observed several times during the measurements (Figure 4). The current spikes were thought to be caused by fusion between liposomes and the Mpp46Ab channel pores and lipid bilayer constructed by the painting method. The channel current through the Mpp46Ab channel pores was very stable, and the pores remained in the open state for at least several minutes (Figure 4). The channel currents were plotted versus the corresponding applied voltage to generate current-voltage relationship graphs.

**Figure 5:**
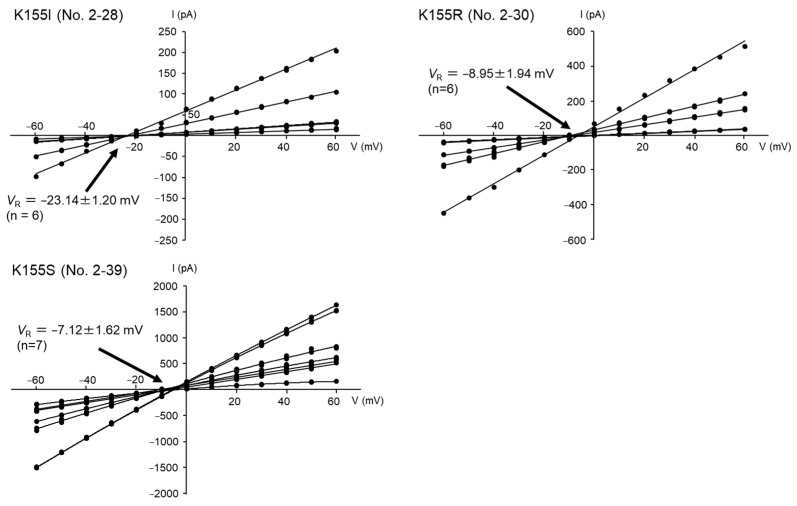
Anion–cation selectivity of channel pores formed by Mpp46Ab mutants K155I, K155R, and K155S. The mean (standard deviation) *V*_R_ was determined using each fitted line.

The current-voltage relationship was generally linear for all three mutants, but the conductance level varied even in measurements for the same mutant (Figure 5). This could have been caused by the fusion of the liposomes with different numbers of Mpp46Ab channel pores to the lipid bilayer in each measurement. Indeed, similar *V*_R_ values were obtained in measurements for the same mutant (Figure 5). The *V*_R_ value for the K155I channel pores was −23.14 ± 1.20 mV (Figure 5), and the *P*_K_/*P*_Cl_ permeability ratio (95% confidence interval) calculated from this *V*_R_ value was 5.80 (5.04–6.56). In contrast, the *V*_R_ value for the K155R channel pores was −8.95 ± 1.94 mV (Figure 5), and the calculated *P*_K_/*P*_Cl_ value of 1.80 (1.57–2.03) was significantly lower than that for the K155I channel pores. Similarly, the *V*_R_ value for the K155S channel pores was −7.12 ± 1.62 mV (Figure 5), with a calculated *P*_K_/*P*_Cl_ value of 1.60 (1.41–1.79). The *P*_K_/*P*_Cl_ value for the K155S channel pores was slightly lower than that for the K155R channel pores, but the difference was not statistically significant. These data thus demonstrated an apparent correlation between cation selectivity (*P*_K_/*P*_Cl_ value) of the Mpp46Ab channel pores and mosquito-larvicidal activity.

## 4. Discussion

Mosquito-larvicidal Mpp46Ab is a pore-forming toxin that produces cation-selective channel pores in artificial lipid bilayers [3,7]. Pore formation is thought to be the central mode of Mpp46Ab action, as the mosquito-larvicidal activity of Mpp46Ab depends on the characteristics of the channel pores. In addition, our previous study suggested that the cation selectivity of the channel pores and resultant mosquito-larvicidal activity can be controlled via mutagenesis targeting the transmembrane β-hairpin region of Mpp46Ab [17].

In the present study, we focused on residue K^155^ within the transmembrane β-hairpin (Figure 1) and constructed a mutant library in which K^155^ was randomly replaced with other amino acids. Following mutagenesis, a total of 161 randomly selected clones were subjected to primary screening using *C. pipiens* mosquito larvae, and 55 clones exhibiting significant toxicity were further analyzed by DNA sequencing (Figure 2). These 55 clones were categorized into 16 types, including the wild-type toxin (Table 1), suggesting that residue K^155^ could be replaced with a variety of other amino acids without diminishing toxicity. However, mutants such as K155D, K155Q, K155P, and K155T were not among the selected clones (Table 1). We considered that the Mpp46Ab channel pores and/or the Mpp46Ab molecule itself of these excluded mutants could have had a structural issue that prevented channel pore formation and/or prevented the toxin from reaching the target site (brush border membrane of the midgut epithelium) as it passed through protease-rich midgut juice. It is also possible that these mutants were not selected in the primary screening by chance.

Bioassays using purified proteins revealed that all 16 Mpp46Ab mutants showed significant toxicity against *C. pipiens* mosquito larvae (Table 2). In particular, the mosquito-larvicidal activity of mutants K155E (LC_50_ = 0.04 μg/mL) and K155I (LC_50_ = 0.09 μg/mL) was significantly higher than that of the wild-type toxin (LC_50_ = 0.51 μg/mL) (Table 2). Mpp46Ab mutant K155E was constructed and characterized previously [13]. Replacement of K^155^ with glutamic acid (K155E) reportedly results in increased cation selectivity of the channel pores and increased mosquito-larvicidal activity. The present study also demonstrated that the K155I channel pores (*P*_K_/*P*_Cl_ = 5.80) were significantly more cation selective than those of the K155R (*P*_K_/*P*_Cl_ = 1.80) and K155S (*P*_K_/*P*_Cl_ = 1.60) mutants examined as controls. According to the colloid-osmotic lysis model, pores formed by insecticidal toxins allow ions and water to enter the target cells, resulting in the disruption of the membrane potential, followed by swelling, lysis, and the eventual death of the host cell [21,22]. The formation of channel pores that are more cation selective may thus enhance the influx of cations and water and facilitate the eventual death of the target cells.

It is of great interest how Mpp46Ab channel pores select ions to pass. It was proposed that the presence of charged amino acid residues within the transmembrane β-hairpin of aerolysin-type PFTs controls the flux of ions through the channel pores [23]. Indeed, aerolysin [24] and ε-toxin [25] form anion-selective channel pores, and their transmembrane β-hairpin contains an excess of positively charged residues. In contrast, the enterotoxin of *C. perfringens* forms cation-selective channel pores, and its transmembrane β-hairpin contains an excess of negatively charged residues [26]. By comparison, in the present study, we observed increased cation selectivity of Mpp46Ab channel pores produced by both the K155E and K155I mutants. Isoleucine is a particularly highly hydrophobic amino acid residue, so the hypothesis may still be controversial. It is at least reasonable to speculate that the charge of residue 155 is not directly involved in determining the cation selectivity of Mpp46Ab channel pores. By replacing K^155^ with glutamic acid or isoleucine, a similar conformational change may be induced in the Mpp46Ab channel pores, particularly in the region that regulates ion selectivity. Mutagenesis experiments targeting residues other than K^155^ within the transmembrane β-hairpin of Mpp46Ab could generate new mutants exhibiting higher toxicity, such as mutants K155E and K155I. In addition, it would be of great interest to identify the residues that are directly involved in determining the ion selectivity within the transmembrane and adjacent regions of Mpp46Ab. In both cases, the strategy combining the introduction of a random mutation with a simplified screening assay using mosquito larvae performed in the present study could facilitate such an investigation. The development of efficient strategies to generate Mpp46Ab mutants with enhanced insecticidal activity may help to prevent or at least delay the onset of resistance in larval mosquito populations.

Mpp46Ab exhibits toxicity against *C. pipiens* mosquito larvae and is a promising candidate for mosquito control. However, many mosquito species, not only *Culex* but also *Anopheles* and *Aedes*, are vectors of serious diseases such as malaria and viral hemorrhagic fevers [27,28]. To use Mpp46Ab as a new mosquito-larvicide, it is important to assess toxicity against *Anopheles* and *Aedes* mosquito larvae. On the other hand, Mpp46Ab exhibits selective cytotoxicity against human leukemic T cells and is a promising candidate for cancer therapy as well. The broad spectrum of Mpp46Ab may be associated with receptor binding, it is of great interest to clarify the determinants of the specificity. Bin toxin produced by *L. sphaericus* is composed of BinA and BinB that act together to intoxicate *Culex* and *Anopheles* mosquito larvae [29,30]. Both BinA and BinB are members of aerolysin-type β-PFTs and share some structural features with Mpp46Aa (PS2Aa) [11]. Potential application to cancer therapy has been assessed and it has been demonstrated that a high concentration of trypsin-activated Bin toxin (particularly BinB subunit) inhibited cell proliferation of human cancer cell lines [31,32]. Comparative analysis, particularly associated with receptor binding, may help to explore the determinants of the specificity of Mpp46Ab as well as Bin toxins. 

## 5. Conclusions

In the present study, we constructed a mutant library in which K^155^ was replaced with other amino acids. Among the mutants selected by primary screening, the toxicity of the K155E and K155I mutants was significantly higher than that of the wild-type toxin. Interestingly, the cation selectivity of K155I channel pores was significantly increased, as previously observed for K155E channel pores. The formation of channel pores that are more cation selective, may enhance the influx of cations and water and facilitate the eventual death of the target cells. Although the mechanism selecting ions to pass is not known, by replacing K^155^ with glutamic acid or isoleucine, a similar conformational change may be induced in the Mpp46Ab channel pores, particularly in the region that regulates ion selectivity. Mutagenesis experiments targeting residues other than K^155^ within the transmembrane β-hairpin of Mpp46Ab could generate new mutants exhibiting higher toxicity. The strategy combining the introduction of a random mutation with a simplified screening assay using mosquito larvae performed in the present study could facilitate investigation.

## Figures and Tables

**Table 1:** Summary of Mpp46Ab mutants constituting the K155rm library.

Amino Acids	Hydropathy Index ^1^	Side Chain (Electrical Charge)	Number of Clones	Name
R	−4.5	Positive	3	K155R
K	−3.9		1	Wild-type
H	−3.2		4	K155H
D	−3.5	Negative	0	-
E	−3.5		2	K155E
N	−3.5	Neutral	2	K155N
Q	−3.5		0	-
P	−1.6		0	-
Y	−1.3		2	K155Y
W	−0.9		3	K155W
S	−0.8		5	K155S
T	−0.7		0	-
G	−0.4		2	K155G
A	1.8		2	K155A
M	1.9		1	K155M
C	2.5		1	K155C
F	2.8		6	K155F
L	3.8		5	K155L
V	4.2		13	K155V
I	4.5		3	K155I

^1^ Values is the hydropathy index defined by Kyte and Doolittles [20].

**Table 2:** Mosquito-larvicidal activity of Mpp46Ab mutants.

Mpp46Ab	Replication (n)	Mosquito-Larvicidal Activity		Representative Clone
		LC_50_ (μg/mL)	95% Confidence Interval	
Wild-type	18	0.51	0.48–0.53	1–2
K155R	6	0.96	0.92–1.00	2–30
K155H	7	0.75	0.71–0.81	2–55
K155E	7	0.04	0.03–0.05	2–1
K155N	5	0.70	0.65–0.74	2–7
K155Y	7	0.55	0.52–0.58	2–48
K155W	6	0.80	0.76–0.85	2–86
K155S	9	0.92	0.87–0.96	2–39
K155G	5	0.50	0.47–0.54	2–58
K155A	9	0.63	0.60–0.66	3–28
K155M	8	0.43	0.40–0.46	3–57
K155C	6	0.70	0.67–0.73	3–33
K155F	6	0.57	0.54–0.61	2–36
K155L	7	0.61	0.57–0.65	3–25
K155V	6	0.87	0.82–0.92	2–3
K155I	7	0.09	0.08–0.10	2–28

## Data Availability

Data are available from the authors upon reasonable request.
